# Supplementary material for: Sleep-dependent reconsolidation after memory destabilization in starlings
Source: Nat Commun. 2018 Aug 6;9:3093. doi: 10.1038/s41467-018-05518-5 (PMC6079047; doi:10.1038/s41467-018-05518-5)
Supplement: Supplementary file 1 — Supplementary Information [file 41467_2018_5518_MOESM1_ESM.pdf]

Supplementary Information

Sleep-dependent reconsolidation after memory destabilization in starlings

Brawn et al.

Supplementary Tables

Supplementary Table 1. Number of trials per session. The number of trials (mean ± standard deviation) run during every training and testing session for experiments 1-4 (a-d). Starlings initiated trials at their own pace during the training and testing sessions. The maximum number of trials allowed was 270 during the training sessions and 30 during the test sessions. The number of training trials tended to be greater in experiments 1 and 3 compared to experiments 2 and 4, but the training sessions for experiments 1 and 3 were 30 minutes longer. The number of test trials completed was comparable for all conditions within each experiment.

a.

Experiment-1	No-Interference	Early-Interference	Late-Interference
**Train-A**	210.8 ± 65.8	209.5 ± 60.3	208.9 ± 64.3
**Test-A1**	29.8 ± 1.3	28.6 ± 4.5	28.4 ± 4.0
**Test-A2**	27.5 ± 5.5	28.2 ± 5.0	29.3 ± 2.3
**Test-A3**	29.3 ± 3.1	29.2 ± 3.6	28.5 ± 4.5
**Test-A4**	28.3 ± 4.6	27.0 ± 6.2	28.7 ± 4.8
**Train-B**	X	193.7 ± 63.6	214.1 ± 67.3
**Test-B**	X	27.7 ± 6.2	29.2 ± 2.8

b.

Experiment-2	No-Interference	Early-Interference	Late-Interference
**Train-A**	162.5 ± 57.8	170.9 ± 61.2	157 ± 54.1
**Test-A1**	28.5 ± 3.7	27.5 ± 5.0	26.6 ± 6.0
**Test-A2**	29.5 ± 2.3	29.4 ± 2.1	25.4 ± 7.5
**Test-A3**	28.5 ± 3.9	29.1 ± 3.5	28.0 ± 4.8
**Test-A4**	27.6 ± 5.4	26.2 ± 6.8	26.9 ± 6.1
**Test-A5**	28.7 ± 4.4	28.8 ± 4.2	28.4 ± 4.9
**Test-A6**	28.0 ± 5.3	27.2 ± 5.2	25.6 ± 7.1
**Test-A7**	30.0 ± 0.0	29.9 ± 0.6	28.9 ± 3.8
**Test-A8**	26.9 ± 7.6	26.7 ± 6.4	26.4 ± 6.5
**Test-A9**	28.6 ± 5.0	29.0 ± 3.6	28.7 ± 3.7
**Train-B**	X	154.6 ± 56.1	173.5 ± 59.6
**Test-B**	X	28.3 ± 4.9	28.1 ± 4.8
**Train-C**	X	163.0 ± 61.8	167.6 ± 54.0
**Test-C**	X	28.7 ± 4.1	28.3 ± 4.0
**Train-D**	X	149.8 ± 54.0	181.0 ± 56.8
**Test-D**	X	28.6 ± 3.9	27.1 ± 6.7

c.

Experiment-3	No-Interference	Interference
**Train-A**	194.8 ± 72.2	199.6 ± 63.0
**Test-A1**	28.1 ± 5.0	29.1 ± 2.9
**Test-A2**	27.8 ± 5.7	29.1 ± 2.6

**Train-B**	X	201.4 ± 65.1
**Test-B**	X	28.9 ± 3.7

d.

**Experiment-4**	**No-Interference**	**Interference**
**Train-A**	146.5 ± 72.5	147.7 ± 60.3
**Test-A1**	26.6 ± 7.1	26.5 ± 7.3
**Test-A2**	27.7 ± 4.4	27.7 ± 6.0
**Train-B**	X	154.6 ± 57.2
**Test-B**	X	26.9 ± 5.7
**Train-C**	X	145.9 ± 54.4
**Test-C**	X	27.3 ± 5.8
**Train-D**	X	144.1 ± 64.4
**Test-D**	X	27.7 ± 4.5

Supplementary Table 2. Task schedule for experimental conditions. The exact timing of the experimental tasks for each condition is presented for experiment-1 (a-c), experiement-2 (d-f), experiment-3 (g-h), and experiment-4 (i-j).

a.

**No-Interference (Experiment-1)**	
**Day-1**	
6:00 A.M. – 9:30 A.M.	Baseline Task
9:30 A.M. – 12:00 P.M.	Train Task-A
12:00 P.M. – 1:00 P.M.	Task-A Test-1
1:00 P.M – 8:00 P.M.	Baseline Task
8:00 P.M. – 6:00 A.M.	Lights Off in Cage (Sleep Period)
**Day-2**	
6:00 A.M. – 12:00 P.M.	Baseline Task
12:00 P.M. – 1:00 P.M.	Task-A Test-2
1:00 P.M. – 5:30 P.M.	Baseline Task
5:30 P.M. – 6:30 P.M.	Task-A Test-3
6:30 P.M. – 8:00 P.M.	Baseline Task
8:00 P.M. – 6:00 A.M.	Lights Off in Cage (Sleep Period)
**Day-3**	
6:00 A.M. – 12:00 P.M.	Baseline Task
12:00 P.M. – 1:00 P.M.	Task-A Test-4
1:00 P.M. – 8:00 P.M.	Baseline Task

b.

**Early-Interference (Experiment-1)**	
**Day-1**	
6:00 A.M. – 9:30 A.M.	Baseline Task
9:30 A.M. – 12:00 P.M.	Train Task-A

12:00 P.M. – 1:00 P.M.	Task-A Test-1
1:00 P.M. – 8:00 P.M.	Baseline Task
8:00 P.M. – 6:00 A.M.	Lights Off in Cage (Sleep Period)
**Day-2**	
6:00 A.M. – 7:30 A.M.	Baseline Task
7:30 A.M. – 10:00 A.M.	Train Task-B
10:00 A.M. – 11:00 A.M.	Test Task-B
11:00 A.M. – 12:00 P.M.	Baseline Task
12:00 P.M. – 1:00 P.M.	Task-A Test-2
1:00 P.M – 5:30 P.M.	Baseline Task
5:30 P.M – 6:30 P.M.	Task-A Test-3
6:30 P.M – 8:00 P.M.	Baseline Task
8:00 P.M. – 6:00 A.M.	Lights Off in Cage (Sleep Period)
**Day-3**	
6:00 A.M. – 12:00 P.M.	Baseline Task
12:00 P.M. – 1:00 P.M.	Task-A Test-4
1:00 P.M. – 8:00 P.M.	Baseline Task

c.

**Late-Interference (Experiment-1)**	
**Day-1**	
6:00 A.M. – 10:00 A.M.	Baseline Task
10:00 A.M. – 12:00 P.M.	Train Task-A
12:00 P.M. – 1:00 P.M.	Task-A Test-1
1:00 P.M – 6:00 P.M.	Baseline Task
6:00 P.M – 7:00 P.M.	Task-A Test-2
7:00 P.M – 8:00 P.M.	Baseline Task
8:00 P.M. – 6:00 A.M.	Lights Off in Cage (Sleep Period)
**Day-2**	
6:00 A.M. – 12:00 P.M.	Baseline Task
12:00 P.M. – 1:00 P.M.	Task-A Test-2
1:00 P.M. – 3:30 P.M.	Train Task-B
3:30 P.M. – 4:30 P.M.	Test Task-B
4:30 P.M. – 5:30 P.M.	Baseline Task
5:30 P.M. – 6:30 P.M.	Task-A Test-3
6:30 P.M. – 8:00 P.M.	Baseline Task
8:00 P.M. – 6:00 A.M.	Lights Off in Cage (Sleep Period)
**Day-3**	
6:00 A.M. – 12:00 P.M.	Baseline Task
12:00 P.M. – 1:00 P.M.	Task-A Test-4
1:00 P.M. – 8:00 P.M.	Baseline Task

d.

No-Interference (Experiment-2)	
**Day-1**	
6:00 A.M. – 10:00 A.M.	Baseline Task
10:00 A.M. – 12:00 P.M.	Train Task-A
12:00 P.M. – 1:00 P.M.	Task-A Test-1
1:00 P.M. – 6:00 P.M.	Baseline Task
6:00 P.M. – 7:00 P.M.	Task-A Test-2
7:00 P.M. – 8:00 P.M.	Baseline Task
8:00 P.M. – 6:00 A.M.	Lights Off in Cage (Sleep Period)
**Days 2-4**	
6:00 A.M. – 12:00 P.M.	Baseline Task
12:00 P.M. – 1:00 P.M.	Task-A:<br>Test-3 (day-2)<br>Test-5 (day-3)<br>Test-7 (day-4)
1:00 P.M. – 6:00 P.M.	Baseline Task
6:00 P.M. – 7:00 P.M.	Task-A:<br>Test-4 (day-2)<br>Test-6 (day-3)<br>Test-8 (day-4)
7:00 P.M. – 8:00 P.M.	Baseline Task
8:00 P.M. – 6:00 A.M.	Lights Off in Cage (Sleep Period)
**Day-5**	
6:00 A.M. – 12:00 P.M.	Baseline Task
12:00 P.M. – 1:00 P.M.	Task-A Test-9
1:00 P.M. – 8:00 P.M.	Baseline Task

e.

Early-Interference (Experiment-2)	
**Day-1**	
6:00 A.M. – 10:00 A.M.	Baseline Task
10:00 A.M. – 12:00 P.M.	Train Task-A
12:00 P.M. – 1:00 P.M.	Task-A Test-1
1:00 P.M. – 6:00 P.M.	Baseline Task
6:00 P.M. – 7:00 P.M.	Task-A Test-2
7:00 P.M. – 8:00 P.M.	Baseline Task
8:00 P.M. – 6:00 A.M.	Lights Off in Cage (Sleep Period)
**Days 2-4**	
6:00 A.M. – 7:00 A.M.	Baseline Task
7:00 A.M. – 9:00 A.M.	Train:<br>Task-B (day-2)<br>Task-C (day-3)<br>Task-D (day-4)
9:00 A.M. – 10:00 A.M.	Test:

	Task-B (day-2)<br>Task-C (day-3)<br>Task-D (day-4)
10:00 A.M. – 12:00 P.M.	Baseline Task
12:00 P.M. – 1:00 P.M.	Task-A:<br>Test-3 (day-2)<br>Test-5 (day-3)<br>Test-7 (day-4)
1:00 P.M. – 6:00 P.M.	Baseline Task
6:00 P.M. – 7:00 P.M.	Task-A:<br>Test-4 (day-2)<br>Test-6 (day-3)<br>Test-8 (day-4)
7:00 P.M. – 8:00 P.M.	Baseline Task
8:00 P.M. – 6:00 A.M.	Lights Off in Cage (Sleep Period)
**Day-5**	
6:00 A.M. – 12:00 P.M.	Baseline Task
12:00 P.M. – 1:00 P.M.	Task-A Test-9
1:00 P.M. – 8:00 P.M.	Baseline Task

f.

**Late-Interference (Experiment-2)**	
**Day-1**	
6:00 A.M. – 10:00 A.M.	Baseline Task
10:00 A.M. – 12:00 P.M.	Train Task-A
12:00 P.M. – 1:00 P.M.	Task-A Test-1
1:00 P.M. – 6:00 P.M.	Baseline Task
6:00 P.M. – 7:00 P.M.	Task-A Test-2
7:00 P.M. – 8:00 P.M.	Baseline Task
8:00 P.M. – 6:00 A.M.	Lights Off in Cage (Sleep Period)
**Days 2-4**	
6:00 A.M. – 12:00 P.M.	Baseline Task
12:00 P.M. – 1:00 P.M.	Task-A:<br>Test-3 (day-2)<br>Test-5 (day-3)<br>Test-7 (day-4)
1:00 P.M. – 3:00 P.M.	Train:<br>Task-B (day-2)<br>Task-C (day-3)<br>Task-D (day-4)
3:00 P.M. – 4:00 P.M.	Test:<br>Task-B (day-2)<br>Task-C (day-3)<br>Task-D (day-4)
4:00 P.M. – 6:00 P.M.	Baseline Task

6:00 P.M. – 7:00 P.M.	Task-A:<br>Test-4 (day-2)<br>Test-6 (day-3)<br>Test-8 (day-4)
7:00 P.M. – 8:00 P.M.	Baseline Task
8:00 P.M. – 6:00 A.M.	Lights Off in Cage (Sleep Period)
**Day-5**	
6:00 A.M. – 12:00 P.M.	Baseline Task
12:00 P.M. – 1:00 P.M.	Task-A Test-9
1:00 P.M. – 8:00 P.M.	Baseline Task

g.

**No-Interference (Experiment-3)**	
**Day-1**	
6:00 A.M. – 9:30 A.M.	Baseline Task
9:30 A.M. – 12:00 P.M.	Train Task-A
12:00 P.M. – 1:00 P.M.	Task-A Test-1
1:00 P.M. – 8:00 P.M.	Baseline Task
8:00 P.M. – 6:00 A.M.	Lights Off in Cage (Sleep Period)
**Day-2**	
6:00 A.M. – 8:00 P.M.	Baseline Task
8:00 P.M. – 6:00 A.M.	Lights Off in Cage (Sleep Period)
**Day-3**	
6:00 A.M. – 12:00 P.M.	Baseline Task
12:00 P.M. – 1:00 P.M.	Task-A Test-2
1:00 P.M. – 8:00 P.M.	Baseline Task

h.

**Interference (Experiment-3)**	
**Day-1**	
6:00 A.M. – 9:30 A.M.	Baseline Task
9:30 A.M. – 12:00 P.M.	Train Task-A
12:00 P.M. – 1:00 P.M.	Task-A Test-1
1:00 P.M. – 8:00 P.M.	Baseline Task
8:00 P.M. – 6:00 A.M.	Lights Off in Cage (Sleep Period)
**Day-2**	
6:00 A.M. – 9:30 P.M.	Baseline Task
9:30 A.M. – 12:00 P.M.	Train Task-B
12:00 P.M. – 1:00 P.M.	Test Task-B
1:00 P.M. – 8:00 P.M.	Baseline Task
8:00 P.M. – 6:00 A.M.	Lights Off in Cage (Sleep Period)
**Day-3**	
6:00 A.M. – 12:00 P.M.	Baseline Task
12:00 P.M. – 1:00 P.M.	Task-A Test-2
1:00 P.M. – 8:00 P.M.	Baseline Task

i.

No-Interference (Experiment-4)	
**Day-1**	**Day-1**
6:00 A.M. – 10:00 A.M.	Baseline Task
10:00 A.M. – 12:00 P.M.	Train Task-A
12:00 P.M. – 1:00 P.M.	Task-A Test-1
1:00 P.M. – 8:00 P.M.	Baseline Task
8:00 P.M. – 6:00 A.M.	Lights Off in Cage (Sleep Period)
**Days 2-4**	**Days 2-4**
6:00 A.M. – 8:00 P.M.	Baseline Task
8:00 P.M. – 6:00 A.M.	Lights Off in Cage (Sleep Period)
**Day-5**	**Day-5**
6:00 A.M. – 12:00 P.M.	Baseline Task
12:00 P.M. – 1:00 P.M.	Task-A Test-2
1:00 P.M. – 8:00 P.M.	Baseline Task

j.

Interference (Experiment-4)	
**Day-1**	
6:00 A.M. – 10:00 A.M.	Baseline Task
10:00 A.M. – 12:00 P.M.	Train Task-A
12:00 P.M. – 1:00 P.M.	Task-A Test-1
1:00 P.M. – 8:00 P.M.	Baseline Task
8:00 P.M. – 6:00 A.M.	Lights Off in Cage (Sleep Period)
**Days 2-4**	
6:00 A.M. – 1:00 P.M.	Baseline Task
1:00 P.M. – 3:00 P.M.	Train:<br>Task-B (day-2)<br>Task-C (day-3)<br>Task-D (day-4)
3:00 P.M. – 4:00 P.M.	Test:<br>Task-B (day-2)<br>Task-C (day-3)<br>Task-D (day-4)
4:00 P.M. – 8:00 P.M.	Baseline Task
8:00 P.M. – 6:00 A.M.	Lights Off in Cage (Sleep Period)
**Day-5**	
6:00 A.M. – 12:00 P.M.	Baseline Task
12:00 P.M. – 1:00 P.M.	Task-A Test-2
1:00 P.M. – 8:00 P.M.	Baseline Task

Supplementary Figures

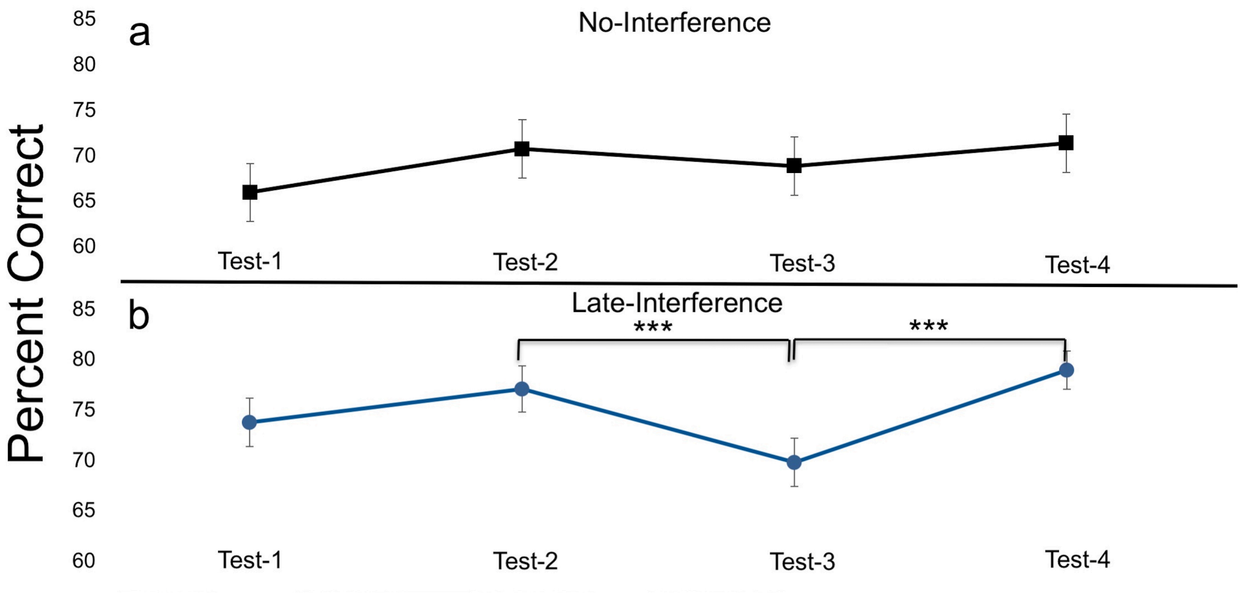

Supplementary Fig. 1. Auditory classification performance for additional starlings in experiment-1. Twenty-four additional starlings completed experiment-1. The mean performance scores from the (a) no-interference and (b) late-interference conditions are shown. The results from the early-interference condition were omitted due to a programming error that resulted in incorrect reward contingencies during the interference task-B test and the task-A test-2. Asterisks indicate significant differences between results at consecutive test times (***$p < 0.001$ after Holm-Bonferroni corrections). Error bars show standard error of the mean.

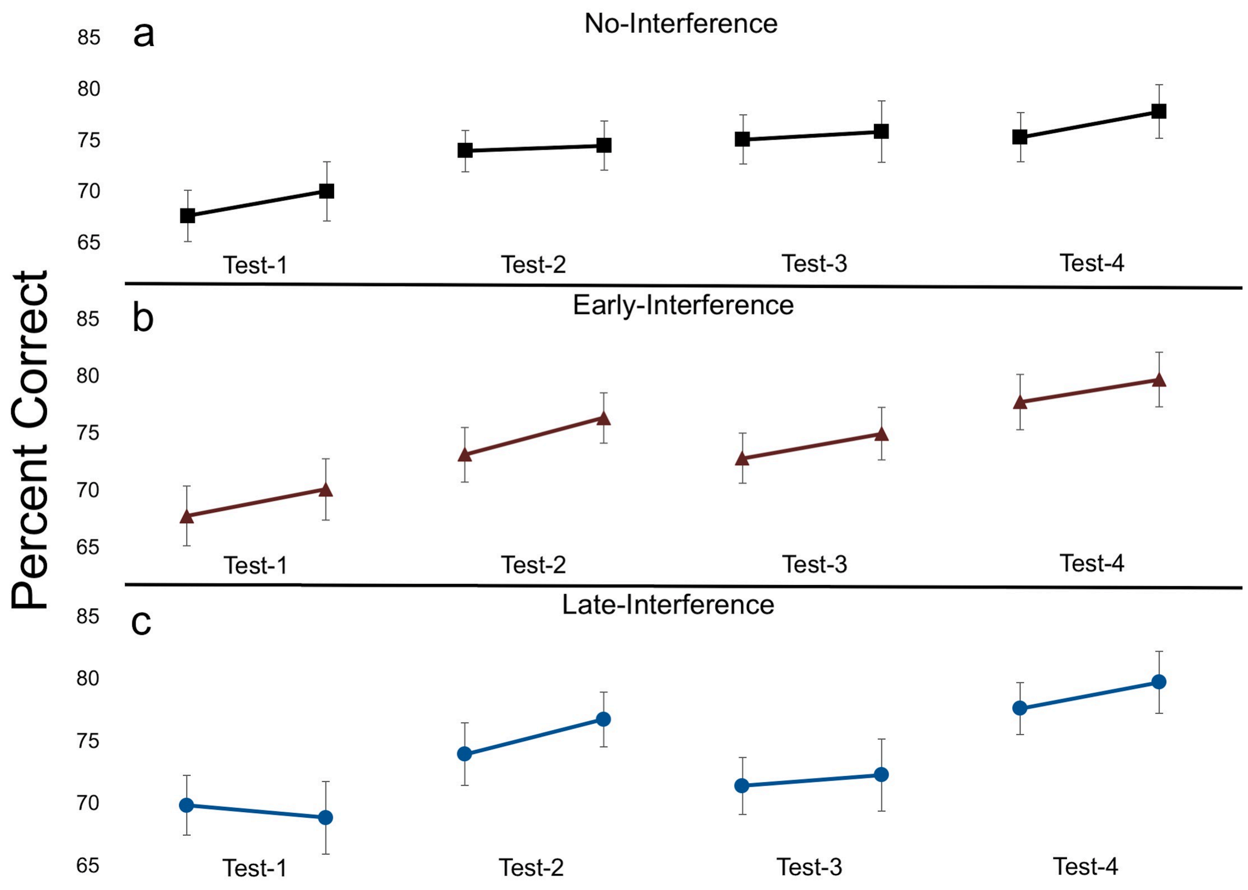

Supplementary Fig. 2. Test session learning dynamics for experiment-1. Classification performance was analyzed separately for the first and second halves of each test session for the (a) no-interference, (b) early-interference, and (c) late-interference conditions to test for within test session learning. Paired t-tests (without correction for multiple comparisons) for each test session did not reveal any significant differences.

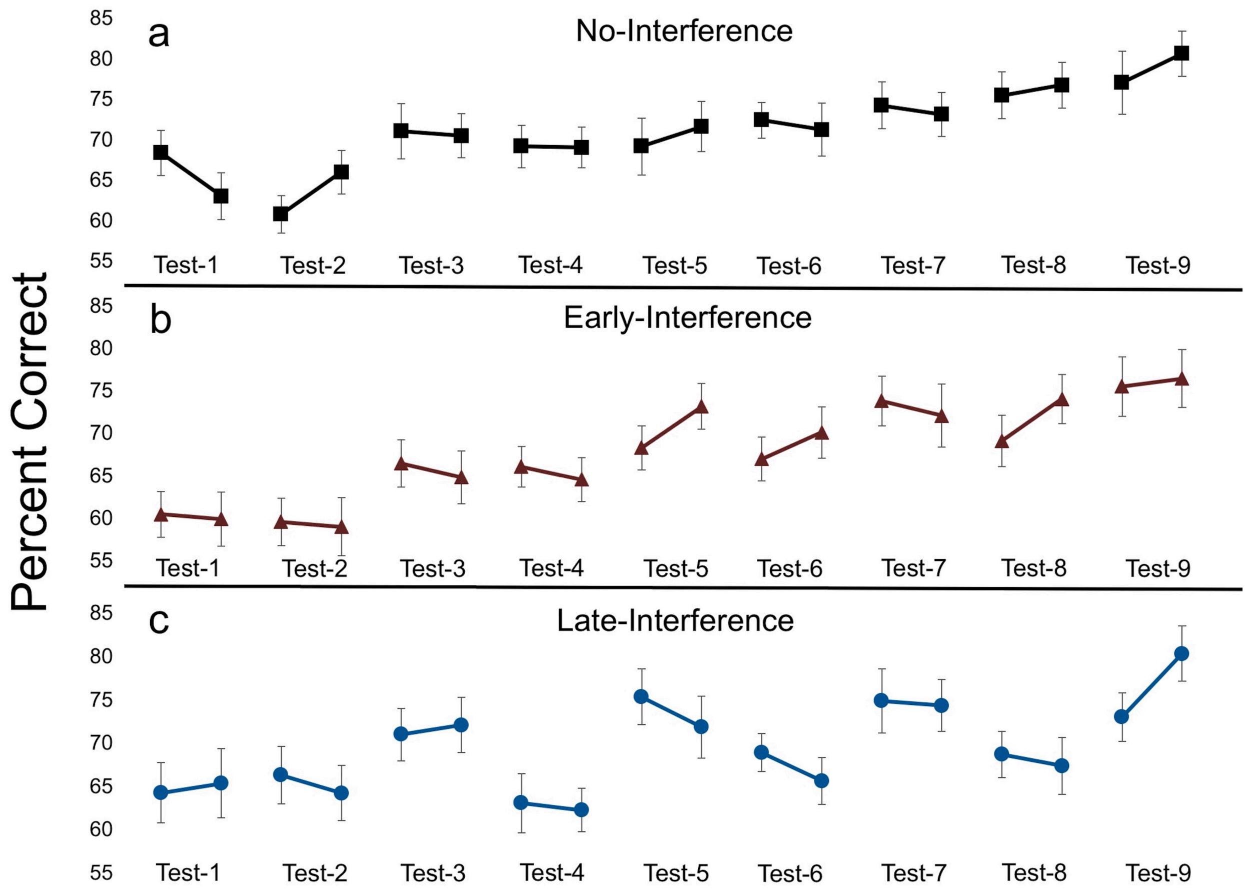

Supplementary Fig. 3. Test session learning dynamics for experiment-2. Classification performance was analyzed separately for the first and second halves of each test session for the (a) no-interference, (b) early-interference, and (c) late-interference conditions to test for within test session learning. Paired t-tests (without correction for multiple comparisons) revealed three significant differences: no-interference Test-2 ($t_{23} = 2.24$, $P = 0.03$), early-interference Test-5 ($t_{23} = 2.43$, $P = 0.02$), and late-interference Test-9 ($t_{23} = 2.83$, $P = 0.01$).

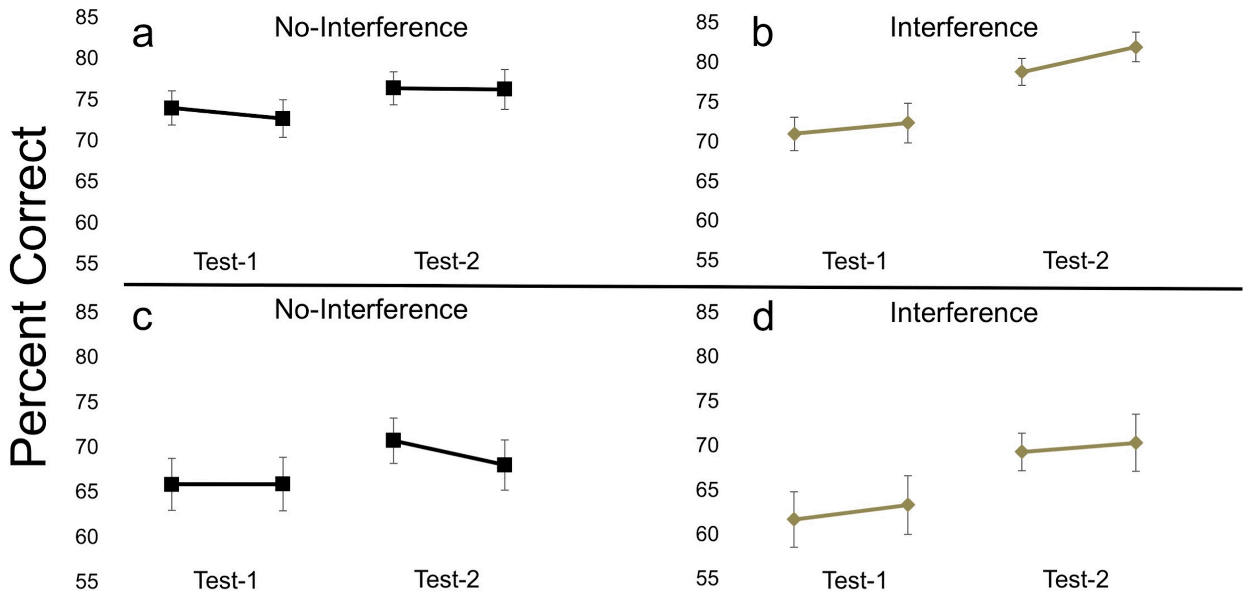

Supplementary Fig. 4. Test session learning dynamics for experiments 3 and 4. Classification performance was analyzed separately for the first and second halves of each test session for the (a) no-interference and (b) interference conditions in experiment-3 and the (c) no-interference and (d) interference conditions in experiment-4 to test for within test session learning. Paired t-tests (without correction for multiple comparisons) revealed one significant difference during the interference Test-2 for experiment-3 (t_{56} = 2.17, P = 0.03).

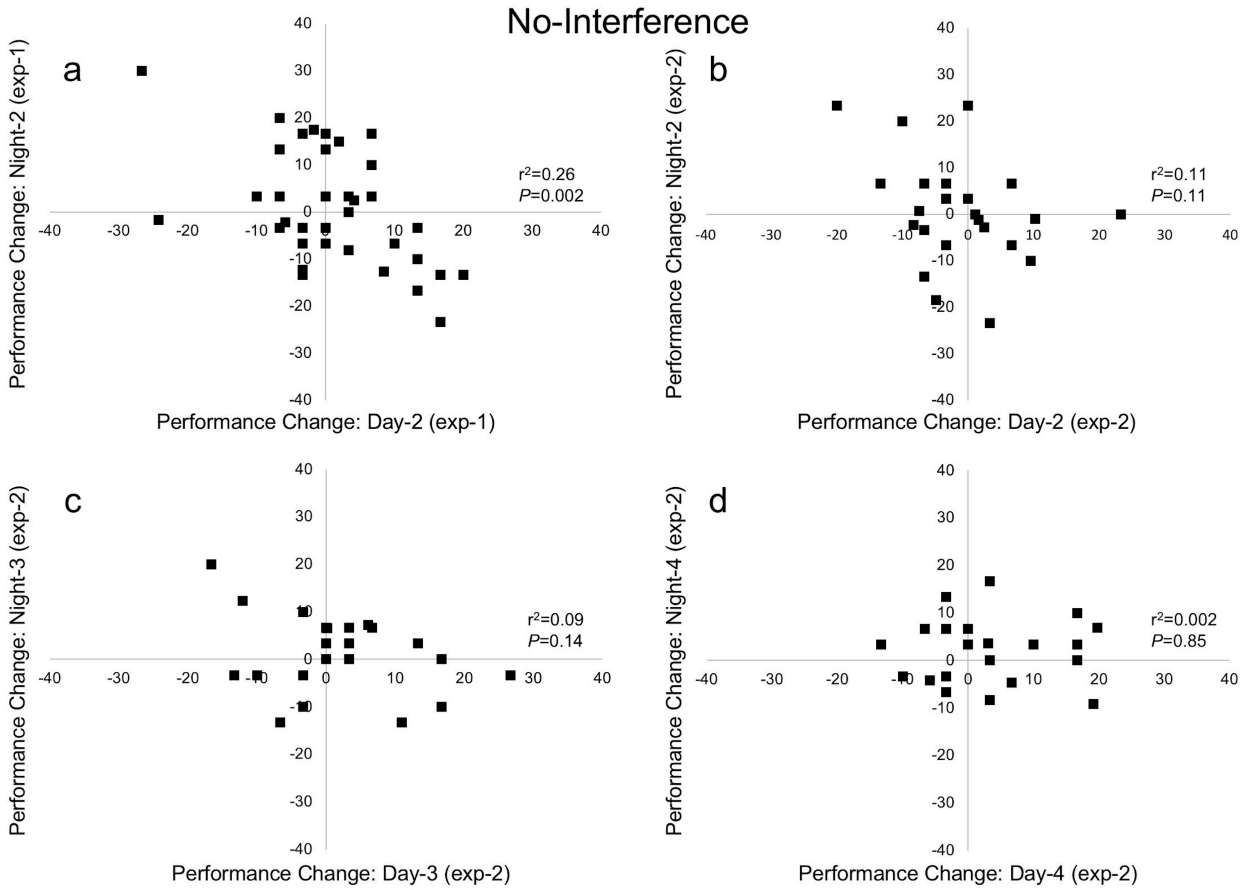

Supplementary Fig. 5. Correlations between daytime performance change and the subsequent nighttime performance change in the No-Interference condition in experiments 1 and 2. The percentage point change in performance across the daytime retention period (12:00 P.M. to 5:30 P.M for experiment-1 or to 6:00 P.M. for experiment-2) is displayed on the x-axis. The percentage point change in performance across the subsequent nighttime retention period (5:30 or 6:00 P.M. to 12:00 P.M. on the next day) is displayed on the y-axis. (a). Day-2 and Night-2 from experiment-1. (b) Day-2 and Night-2 from experiment-2. (c) Day-3 and Night-3 from experiment-2. (d) Day-4 and Night-4 from experiment-2.

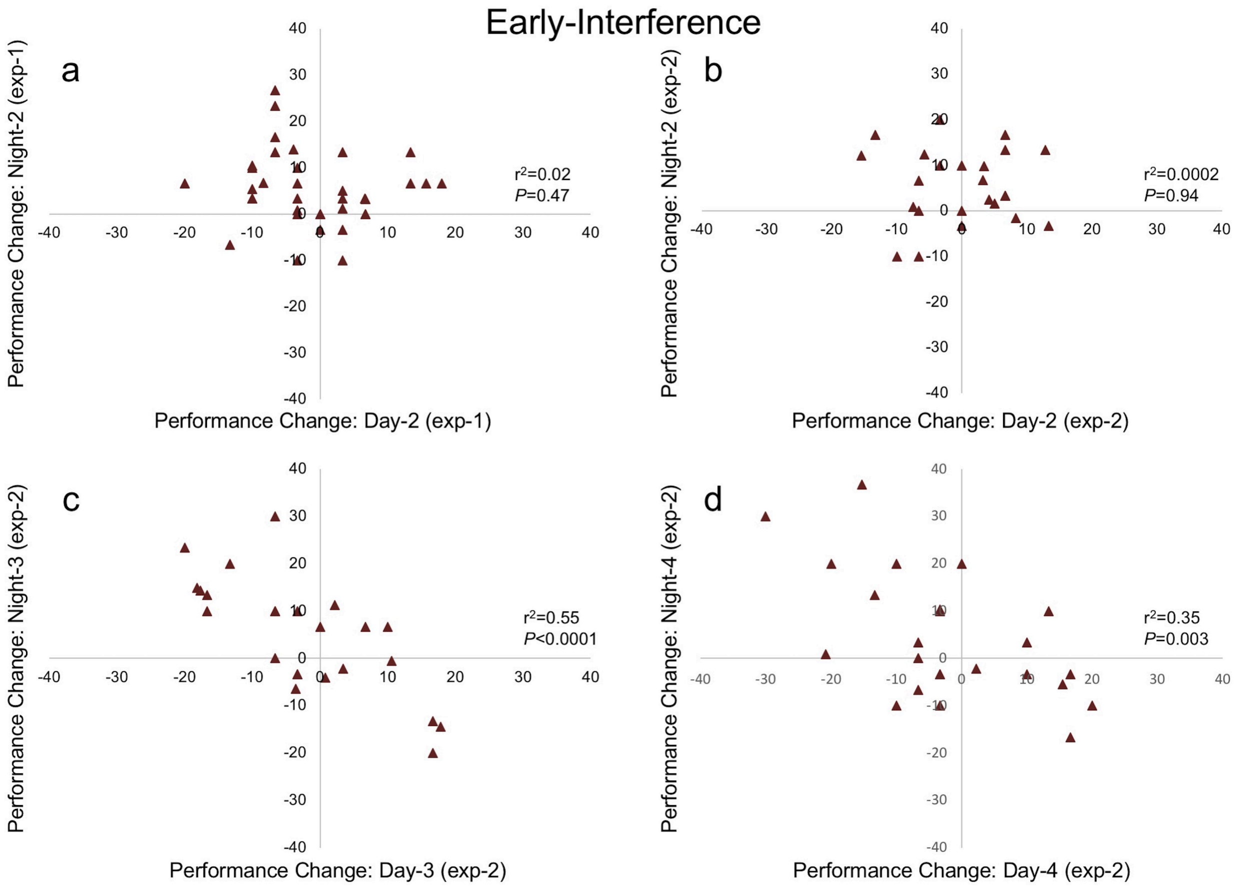

Supplementary Fig. 6. Correlations between daytime performance change and the subsequent nighttime performance change in the Early-Interference condition in experiments 1 and 2. The percentage point change in performance across the daytime retention period (12:00 P.M. to 5:30 P.M for experiment-1 or to 6:00 P.M. for experiment-2) is displayed on the x-axis. The percentage point change in performance across the subsequent nighttime retention period (5:30 or 6:00 P.M. to 12:00 P.M. on the next day) is displayed on the y-axis. (a). Day-2 and Night-2 from experiment-1. (b) Day-2 and Night-2 from experiment-2. (c) Day-3 and Night-3 from experiment-2. (d) Day-4 and Night-4 from experiment-2.

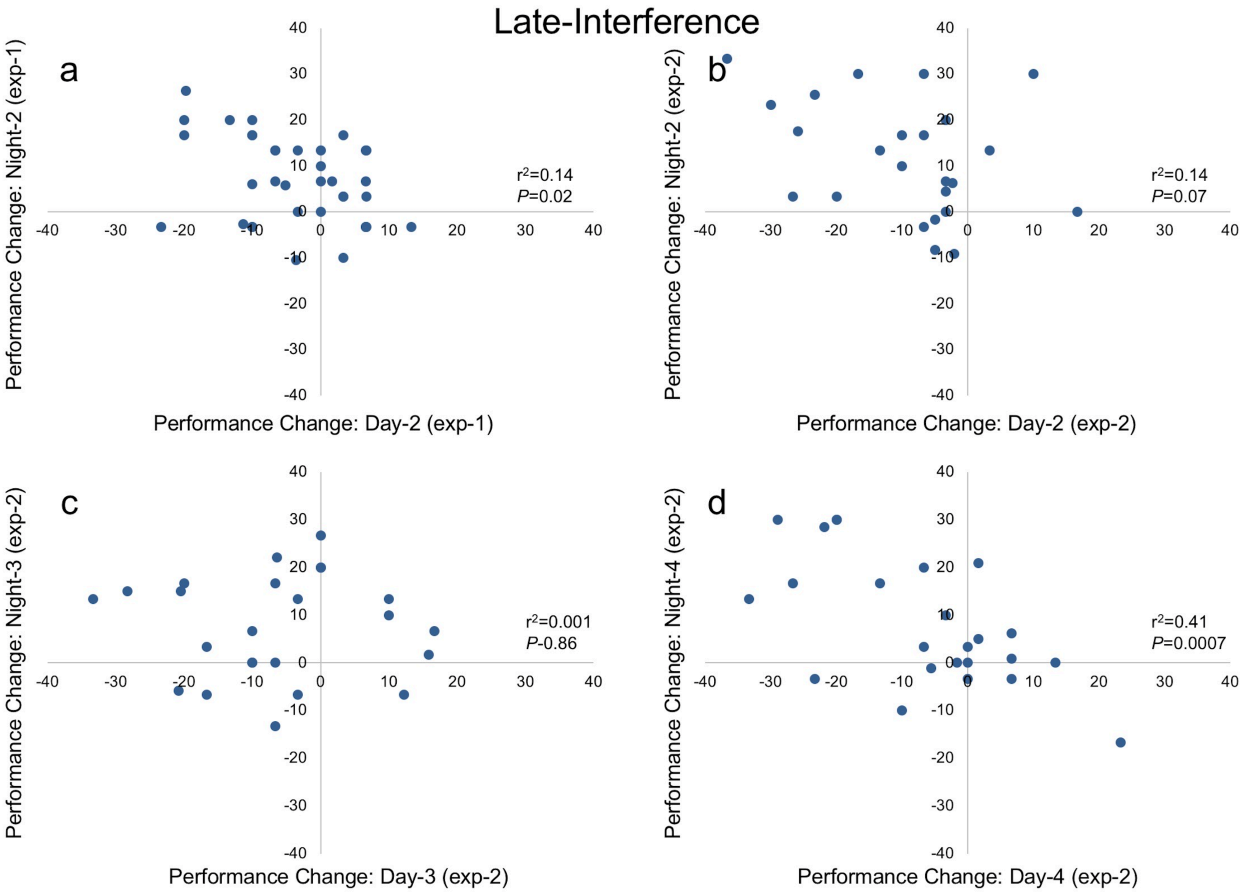

Supplementary Fig. 7. Correlations between daytime performance change and the subsequent nighttime performance change in the Late-Interference condition in experiments 1 and 2. The percentage point change in performance across the daytime retention period (12:00 P.M. to 5:30 P.M for experiment-1 or to 6:00 P.M. for experiment-2) is displayed on the x-axis. The percentage point change in performance across the subsequent nighttime retention period (5:30 or 6:00 P.M. to 12:00 P.M. on the next day) is displyed on the y-axis. (a). Day-2 and Night-2 from experiment-1. (b) Day-2 and Night-2 from experiment-2. (c) Day-3 and Night-3 from experiment-2. (d) Day-4 and Night-4 from experiment-2.

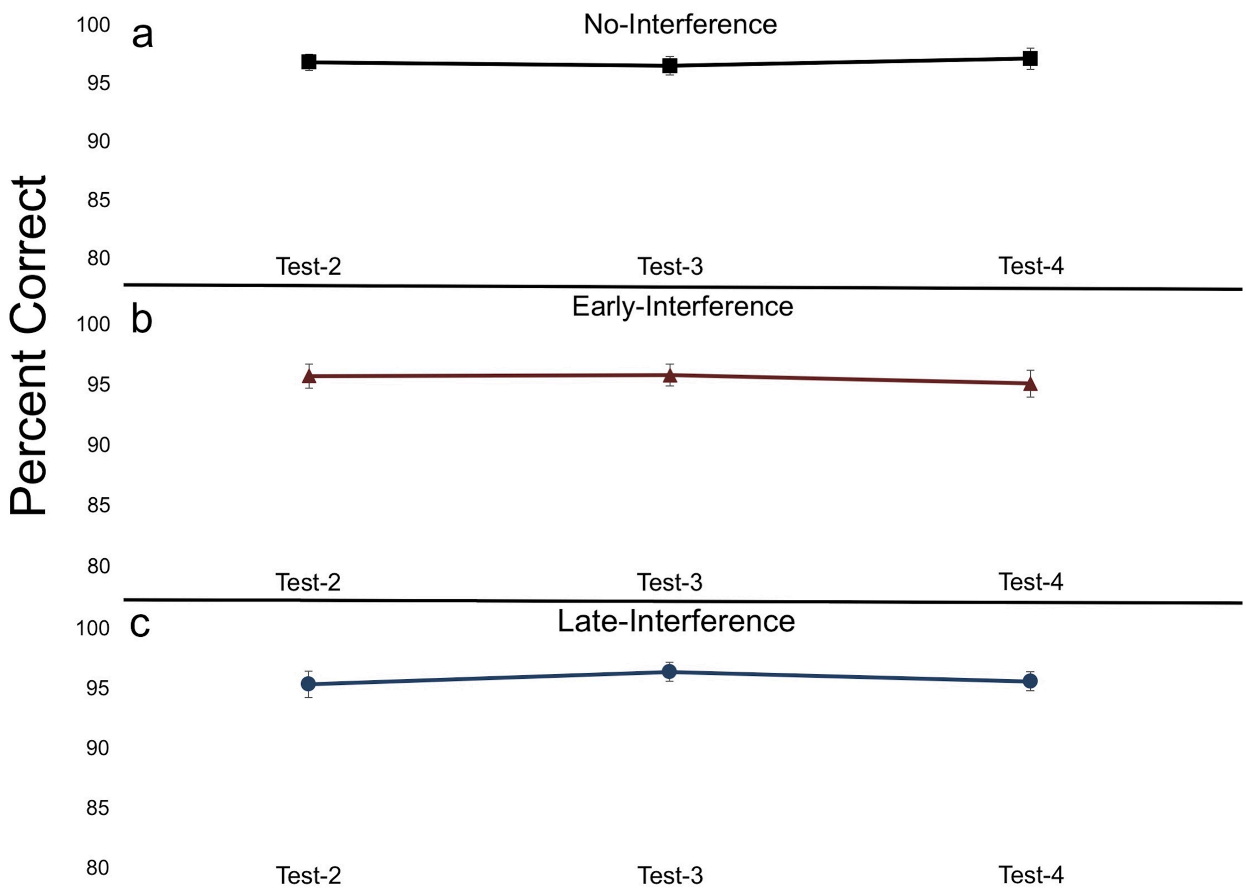

Supplementary Fig. 8. Auditory classification performance on baseline task for experiment-1. Performance on the 50 baseline trials preceding test sessions 2-4 were analyzed to control for circadian factors on performance since tests 2 and 4 (12:00 P.M.) were conducted at a different time of day than test-3 (5:30 P.M.). Baseline performance before test-1 was not analyzed because test-1 was preceded by a 2.5-hour task-A training session rather than the baseline task. (a) Baseline classification task performance in the no-Interference condition. (b) Baseline classification task performance in the early-interference condition. (c) Baseline classification task performance in the late-interference condition. Baseline task performance did not differ depending on time of day ($F_{(2,70)} = 0.13$; $P = 0.88$).

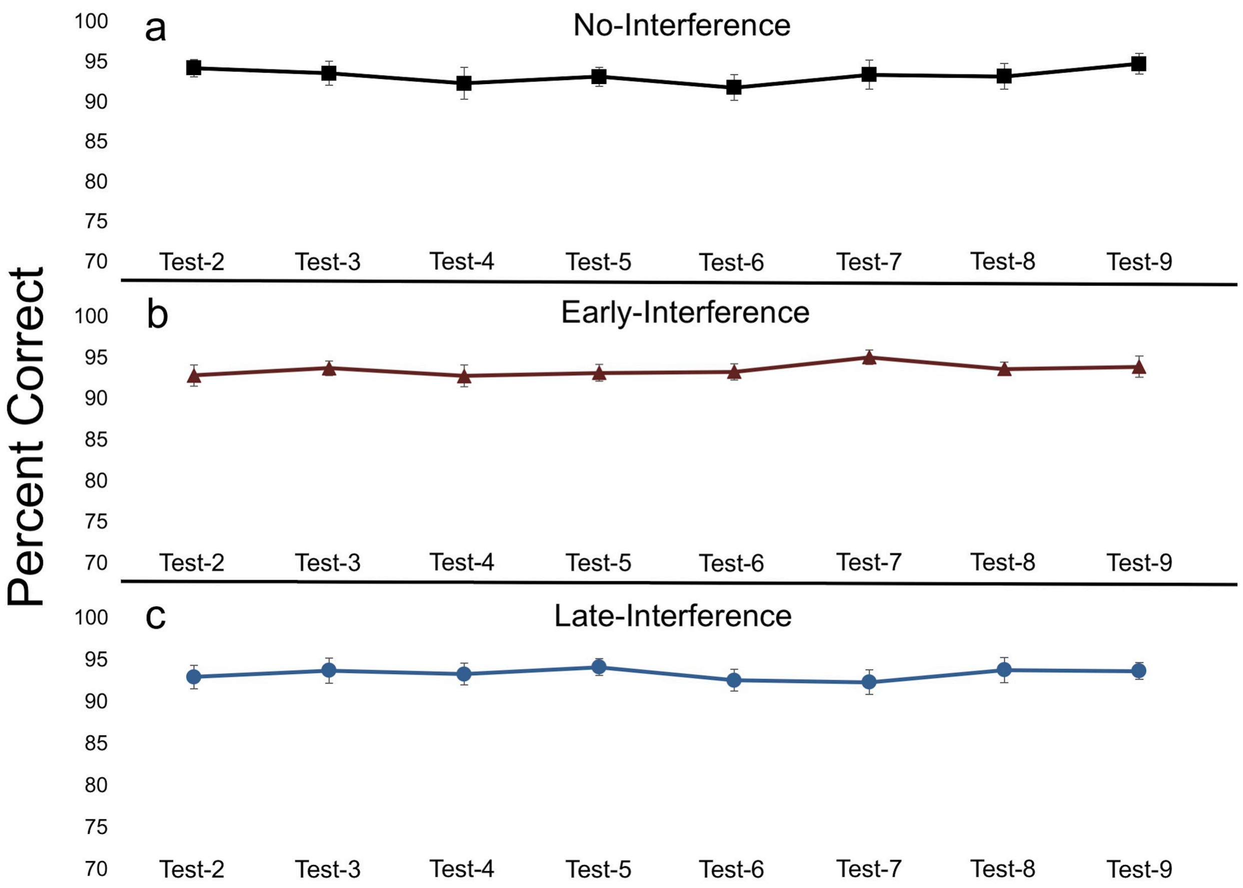

Supplementary Fig. 9. Auditory classification performance on baseline task for experiment-2. Performance on the 50 baseline trials preceding test sessions 2-9 were analyzed to control for circadian factors on performance since tests 3, 5, 7, and 9 (12:00 P.M.) were conducted at a different time of day than tests 2, 4, 6, and 8 (6:00 P.M.). Baseline performance before test-1 was not analyzed because test-1 was preceded by a 2-hour task-A training session rather than the baseline task. (a) Baseline classification task performance in the no-Interference condition. (b) Baseline classification task performance in the early-interference condition. (c) Baseline classification task performance in the late-interference condition. Baseline task performance did not differ depending on time of day ($F_{(7,161)} = 0.56$; $P = 0.79$).
